# Phylogenomic analysis supports *Mycobacterium tuberculosis* transmission between humans and elephants

**DOI:** 10.3389/fvets.2023.1133823

**Published:** 2023-05-26

**Authors:** Rajesh Man Rajbhandari, Rajindra Napit, Prajwol Manandhar, Roji Raut, Anupama Gurung, Ajit Poudel, Nisha Shrestha, Amir Sadaula, Dibesh Karmacharya, Christian Gortázar, Paulo Célio Alves, José de la Fuente, João Queirós

**Affiliations:** ^1^Departamento de Biologia, Faculdade de Ciencias da Universidade do Porto, Porto, Portugal; ^2^CIBIO, Centro de Investigação em Biodiversidade e Recursos Genéticos, InBIO Laboratório Associado—BIOPOLIS Program in Genomics, Biodiversity and Land Planning, Campus de Vairão, Universidade do Porto, Vairão, Portugal; ^3^SaBio, Instituto de Investigación en Recursos Cinegéticos, IREC (UCLM and CSIC), Ciudad Real, Spain; ^4^Center for Molecular Dynamics Nepal, Kathmandu, Nepal; ^5^National Trust for Nature Conservation, Biodiversity Conservation Center, Chitwan, Nepal; ^6^School of Biological Sciences, Faculty of Science, The University of Queensland, Brisbane, QLD, Australia; ^7^EBM, Estação Biológica de Mértola, Praça Luís de Camões, Mértola, Portugal; ^8^Department of Veterinary Pathobiology, Center for Veterinary Health Sciences, Oklahoma State University, Stillwater, OK, United States

**Keywords:** *Elephas maximus*, whole-genome sequencing, one health, tuberculosis, zooanthroponosis

## Abstract

**Introduction:**

Tuberculosis is an infectious disease caused by a group of acid-fast bacilli known as *Mycobacterium tuberculosis* complex (MTC), which has a major impact on humans. Transmission of MTC across the human-animal interface has been demonstrated by several studies. However, the reverse zoonotic transmission from humans to animals (zooanthroponosis) has often been neglected.

**Methods:**

In this study, we used Nanopore MinION and Illumina MiSeq approaches to sequence the whole genome of *M. tuberculosis* strains isolated from two deceased Asian elephants (*Elephas maximus*) and one human in Chitwan, Nepal. The evolutionary relationships and drug resistance capacity of these strains were assessed using the whole genome data generated by the stand-alone tool Tb-Profiler. Phylogenomic trees were also constructed using a non-synonymous SNP alignment of 2,596 bp, including 94 whole genome sequences representative of the previously described *M. tuberculosis* lineages from elephants worldwide (lineages 1 and 4) and from humans in Nepal (lineages 1, 2 and 3).

**Results and Discussion:**

The new genomes achieved an average coverage of 99.6%, with an average depth of 55.67x. These *M. tuberculosis* strains belong to lineage 1 (elephant DG), lineage 2 (elephant PK) and lineage 4 (human), and none of them were found to have drug-resistant variants. The elephant-derived isolates were evolutionarily closely related to human-derived isolates previously described in Nepal, both in lineages 1 and 2, providing additional support for zooanthroponosis or bidirectional transmission between humans and elephants. The human-derived isolate clustered together with other published human isolates from Argentina, Russia and the United Kingdom in the lineage 4 clade. This complex multi-pathogen, multi-host system is challenging and highlights the need for a One Health approach to tuberculosis prevention and control at human-animal interface, particularly in regions where human tuberculosis is highly endemic.

## 1. Introduction

Tuberculosis (TB) is a significant global burden and is widely reported to be a major public health and economic problem, costing the world $617 billion between 2000 and 2015 and projected to cost $1 trillion between 2015 and 2030 (1). It is the second leading cause of death after COVID-19, with an estimated 10 million cases worldwide and 1.5 million deaths in 2020 (2). Drug-resistant TB is also a major threat to global disease control (3). It is a major contributor to global antimicrobial resistance (4). In 2021, 450,000 cases of rifampicin-resistant and multidrug-resistant (MDR/MDR-TB) TB were forecasted worldwide, of which 191,000 died (5). In Nepal, the number of cases of drug-resistant TB is estimated to be around 1,500 per year (6).

Human and animal tuberculosis is caused by a group of closely related acid-fast bacilli known as the *Mycobacterium tuberculosis* complex (MTC) (7, 8). The MTC includes several species of mycobacteria, namely *M. tuberculosis*, *Mycobacterium bovis* (Bacillus Calmette–Guerin), *Mycobacterium africanum*, *Mycobacterium caprae*, *Mycobacterium microti*, *Mycobacterium canettii* and *Mycobacterium pinnipedii* (9). *M. tuberculosis* is the human-adapted variant and is the major cause of human TB (9). However, most publications on emerging infectious agents such as MTC often focus on their zoonotic origin, but comparatively fewer reports are published on possible zooanthroponosis and the human origin of infectious diseases affecting domestic or wild animals (10). Zooanthroponosis, is an important ongoing debate regarding the transmission of pathogens from humans to animals. Evidence from a global survey showed that humans are capable of transmitting at least 21 bacterial, 12 viral and 7 fungal pathogens to animals (10). Although there is evidence that pathogens can be transmitted from humans to animals, most reported cases involve captive or domestic animals (11). Influenza is one of the best known examples of bidirectional transmission between humans and domestic pigs (12). The emergence and re-emergence of these pathogens has important implications for human and animal health (13). This risk appears to be even greater when bidirectional transmission between humans and animals is frequent, as in the case of TB in elephants (14).

Tuberculosis in elephants has been reported worldwide, although most reported cases involve captive elephants with *M. tuberculosis*, the ethological agent of human TB (15–19). In countries such as Nepal, where there is a high prevalence of active *M. tuberculosis* infection in humans (117,000 people living with TB; 20) and a large elephant population (more than 200 captive and 200–250 wild elephants; 21), this issue is particularly relevant. Elephant-human interactions are particularly high in regions where captive elephants are used for ecotourism and patrolling protected areas, such as Chitwan National Park. This poses a serious risk of zooanthroponosis and disease to both captive and wild elephant populations, as well as a risk to humans given the possibility of bidirectional transmission (15, 22). A recent study in four national parks in Nepal showed a high seroprevalence (21.56%) of TB in captive elephants, with most cases detected in Chitwan National Park (23). In addition, some evidence of zooanthroponosis has been reported from previous studies in Nepal that also isolated *M. tuberculosis* from diseased elephants using multi-locus variable number of tandem repeats (MLVA) (15, 24), large sequence polymorphism (24) or whole genome data (25). However, the latter study lacks a phylogenetic analysis of the two *M. tuberculosis* genomes (26) and their integration with previously published data (25).

In this study, a whole genome sequencing approach was used to genotype *M. tuberculosis* isolates from a human and two deceased elephants in Chitwan, Nepal. These new data, combined with genomic data from elephants and human isolates available in the literature, allowed a comprehensive evolutionary study of *M. tuberculosis* in Nepal. These results contribute to the understanding of the possible bidirectional transmission of this pathogen at human-elephant interface.

## 2. Methods and materials

### 2.1. Study area

**Figure 1:**
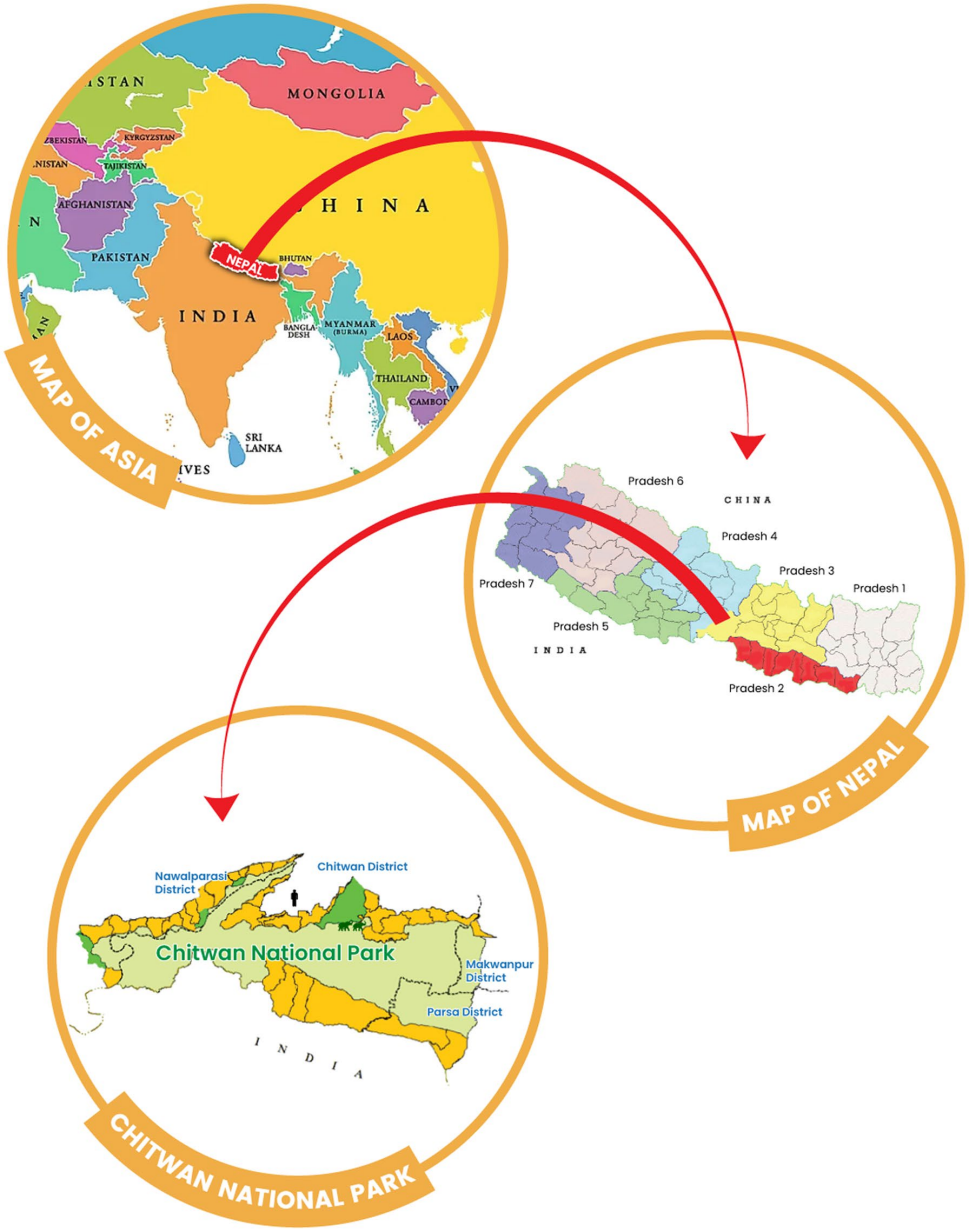
Geographical map of Nepal showing the location of Chitwan National Park (light green) and the origin of the human and elephant samples used in this study (icons). Forest communities around Chitwan National Park are shown in dark green.

Chitwan district is located in the southwestern part of Bagmati Province in Nepal. It covers an area of 2,238.39 km^2^ and had a human population of 719,859 in 2022. Nepal’s first national park, Chitwan National Park (CNP), is located in this district (Figure 1) and was established in 1973 (27). The park covers an area of 932 km^2^ and is located in the subtropical Terai lowlands of south-central Nepal. It lies in a river valley basin or dun, along the floodplains of the Rapti, Reu and Narayani rivers. The Chitwan valley consists of tropical and subtropical forests. Sal forest covers 70% of the park. Sal leaves are used locally for plates in festivals and religious offerings, and are also known for their Ayurvedic use in the treatment of wounds, coughs and other ailments (28). Grasslands cover 20 per cent of the park. There are more than 50 different species of grasses, including elephant grass (*Saccharum* spp), known for its immense height. The climate is mainly dominated by the summer monsoon, and the valley experiences three distinct seasons each year: winter, summer and monsoon.

Chitwan National Park is regarded as one of the best national parks for wildlife viewing in Asia (29). It is home to 68 species of mammals, more than 576 species of birds, 49 species of reptiles and amphibians, 120 species of fish and several species of invertebrates that contribute significantly to ecosystem processes in the region. The reserve is home to many of Nepal’s charismatic wildlife species, including the one-horned rhinoceros (*Rhinoceros unicornis*), Bengal tiger (*Panthera tigris*), leopard (*Panthera pardus*) and Asian elephant (*Elephas maximus*), among others (29, 30). Of the various recreational activities available in the park, elephant safaris are the most popular, especially in and around the buffer zones. Elephants are also used by the government for transport and patrolling the park. Buffer zones in the CNP were established in 1996 with the aim of involving people in the management of park resources for the conservation of biodiversity and the livelihoods of buffer zone communities (31). The buffer zone covers an area of 750 km^2^ and is home to approximately 45,516 houses and 2,60,352 people (32). Buffer zones are primarily designed to create human-wildlife coexistence by providing an ecological and socio-economic buffer for communities, and in these areas, where wildlife and humans share the same landscape in close proximity, their interaction is very obvious (33).

### 2.2. Sample collection

Samples were collected from two deceased adult (>30 years) elephants in Sauraha (Elephant PK and Elephant DG), Chitwan, owned by the government, and used for tourism, grass collection and patrolling. Sauraha is a village at the eastern entrance to Chitwan National Park, near the East Rapti River. These animals had died in captivity and at necropsy showed granulomatous lesions in the lungs compatible with tuberculosis. Tissue samples from lung biopsies (with lesions) were taken from both elephants. The human sputum sample (No. 52G111) collected in Chitwan as part of a previous study (20) was also genotyped, although it was not closely related to the diseased elephants. It was included to understand the evolutionary relationship with the *M. tuberculosis* strains isolated from elephants, as it was collected from the same region.

### 2.3. Molecular confirmation of *Mycobacterium tuberculosis* infection

The elephant lung tissue samples (PK and DG) and human sputum samples (52G111) were sent to GENETUP (German Nepal Tuberculosis Project microbiology lab) for culture confirmation of *M. tuberculosis* as described in (34). The heat-killed cultures were then resuspended in 100 μL of PBS for DNA extraction. The DNA extraction was performed using a DNA-SorB kit according to the manufacturer’s instructions (Sacace Biotechnologies, Italy). Briefly, 300 μL of the lysis solution was mixed with 100 μL of the heat lysed *M. tuberculosis* PBS solution and incubated at 60°C for 5 min. The samples were centrifuged at 15000 g for 5 min to separate the cell debris and DNA in the supernatant. The supernatant was then mixed with 20 μL of the sorbent to bind the DNA to the sorbent. The sorbent was washed twice, dried at 65°C for 5 min and eluted with 25 μL of DNA eluent. Elution was further enhanced by incubating the tubes with DNA eluent at 65°C for 5 min and periodically vortexing. The extracted DNA was then quantified using Qubit (Thermo Fisher Scientific, USA) and *M. tuberculosis* infection was diagnosed using the specific real-time PCR (35).

### 2.4. Whole-genome sequencing of elephant and human *Mycobacterium tuberculosis* isolates

Two complementary whole genome sequencing approaches were used in this study: Illumina short reads and MinION long reads. In the case of Illumina short reads, genomic DNA from the heat-killed cultures of *M. tuberculosis* was purified using AMPure beads (Beckman Coulter, United States) (1:1), quantified using Qubit (Thermo Fisher Scientific, United States) and normalised to 0.2 ng/μL. The normalised samples were tagged and indexed using the Nextera Indexing Kit (Illumina, United States). The indexed library was then purified using (0.8X) AMPure beads and quantified using HS kit (Thermo Fisher Scientific, United States). Each library was analysed using a bioanalyzer and normalised to a final concentration of 2 nM. The three normalised libraries were then pooled in equal volumes of 5 μL to give a final pooled library of 2 nM. The pooled library was spiked with 5% of 2 nM PhiX and denatured with NaOH. The denatured library was diluted to 20 pM, then 10 pM using hybridisation buffer, and the final 10 pM library was loaded onto the Illumina MiSeq platform using the MiSeq Reagent Kit V2 300-cycles (Illumina, United States), with an estimated coverage of approximately100× per sample.

For long-read sequencing in Nanopore MinION, normalised genomic DNA (0.2 ng/μL) from the heat-killed cultures of *M. tuberculosis* isolated from the two elephants was also used for long-read sequencing in Nanopore MinION, using the Nextera XT Library Preparation Kit (Illumina, USA). The 20 μL of tagged genomic DNA was end-repaired using Ultra II End Prep Buffer and Ultra II End Prep Enzyme (NEB, United Kingdom) according to the manufacturer’s instructions. The end-repaired DNA was then subjected to Nanopore DNA preparation using Ligation sequencing kit (Oxford Nanopore, United Kingdom) with PCR barcoding expansion kit (Oxford Nanopore, United Kingdom). The final library was purified using 0.4X AMPure beads and Long Fragment Buffer (LFB) to obtain the eluate in 15 μL elution buffer. The adapter-ligated library was quantified using the Qubit Fluorometer and approximately 15 fm of the total library was finally loaded onto the MinION for sequencing using 15 μL of sequencing buffer and 10 μL loading beads.

### 2.5. Lineage and multi drug resistance status determination

Tb-Profiler version 4.1.0, a stand-alone tool (36), was used for lineage identification and drug resistance assessment of *M. tuberculosis* isolates from whole genome sequences. The tool accepts raw data from both MinION Nanopore and Illumina MiSeq sequences as well as BAM and FASTA files. It is a command-line tool that can use whole-genome data to predict lineages and identify 21 types of drug resistance (based on small variants and large deletions associated with drug resistance).

### 2.6. Processing and analysis of Illumina and Nanopore sequence data

Quality control and filtering of Illumina reads was performed using Fastp v0.20 (37), while NanoFilt v2.6.0 (38) was used to filter Nanopore reads. After quality filtering, the Illumina and Nanopore reads were mapped separately to the *M. tuberculosis* reference genome (GenBank accession NC_000962.3) using BWA v0.7.17 (39). SAMtools v1.15 (40) was used to convert the SAM files of the mapped reads from both Illumina and Nanopore to BAM files, and to sort and index the BAM file reads using the view, sort and index commands. SAMtools merge was then used to merge the index and sorted BAM files into a single BAM file containing both long and short reads for each sample (40).

### 2.7. Phylogenomic analysis

To performe a comparative phylogenomic analysis of *M. tuberculosis* isolates, we selected a sequence dataset comprising 94 full-length *M. tuberculosis* whole genomes, including data from human isolates in Nepal (*n* = 8), and a genome of *M. africanum* to use as an outgroup. These genomes were from 33 different countries and the four different geographical lineages previously described from elephants worldwide (lineages 1 and 4) and from humans in Nepal (lineages 1, 2 and 3), and they were retrieved from the NCBI Sequence Read Archive (SRA) database (Supplementary Table S1). The *prefetch* command in the SRA toolkit v2.11.0 (41) was used to download all whole-genome sequence files of our dataset in SRA format, which were later converted to FASTQ format using the *fastq-dump* command. Fastp v0.20 was used for quality control and read filtering (37). Read mapping to the reference genome (GenBank accession: NC_000962.3) was performed using BWA v0.7.17 (39). SAMtools v1.15 was used to convert the SAM files of the mapped reads to BAM files, and then to sort and index the BAM file reads (40). The SAMtools *mpileup* command generated a text pileup output, summarizing the base calls of the aligned reads to the reference sequence. The consensus call was performed using the *call* command in BCFtools v1.10 (40), which produced a VCF file that was converted to a FASTQ file using the *vcf2fq* function in the *vcfutils.pl* script (42). Seqtk v1.3 (42) was used to convert the FASTQ files to FASTA files using the *seq* command with the masking of bases with quality less than 20.

Variant calling for all the downloaded *M. tuberculosis* sequences and three *M. tuberculosis* isolates from this study was performed using PhaME v1.0.4 (43), with the H37Rv genome (GenBank accession: NC_000962.3) as the reference. The PhaME analysis tool was used to remove repeats from each genome in the dataset, and genome alignment to the reference genome was performed using the nucmer2 tool from MUMmer v3.0, as described in the pipeline (43). The output genome alignments were filtered, and SNP tables were generated for each alignment using the *delta-filter* and *show-snps* utilities, respectively, both in MUMmer v3.0. The PhaME pipeline then collated the alignment files to produce a core genome alignment and output a multiple alignment FASTA file (~200 kbp) with the variant sites. This was further filtered with SNP-sites to remove non-informative synonymous sites, resulting in a final non-synonymous SNP alignment dataset of 2,596 base pairs.

Using the non-synonymous SNP alignment data, phylogenomic analysis was performed using maximum likelihood and Bayesian inference. For maximum likelihood, the best substitution models and likelihood trees were evaluated using IQTREE v1.6.11 (44) with 500 bootstrap replicates and default parameters. Similarly, for Bayesian inference, the best nucleotide substitution model, TVM, was selected based on the Bayesian Information Criterion score in jModeltest2 v2.1.8 (45, 46), followed by tree reconstruction in MrBayes v3.2.7 (47) with 2,000,000 iterations, discarding 25% as burn-in.

## 3. Results

### 3.1. Molecular screening results

The two heat-killed culture samples from elephants yielded 1.07 ng/μL and 0.35 ng/μL DNA for DG and PK, respectively, allowing confirmation of *M. tuberculosis* by real-time PCR. The *M. tuberculosis* isolated from the human sample 52G11 was already a confirmed cultured *M. tuberculosis* with a DNA concentration of 2.34 ng/μL.

### 3.2. TB-profiler results

Lineage analysis using TB-Profiler showed that the three isolates belonged to three different *M. tuberculosis* lineages (Table 1). The elephant isolates DG and PK belong to lineage/sub-lineage 1.2.2.2 and 2.2, respectively, whereas the human isolate 52G111 belongs to lineage/sub-lineage 4.1.2.1. None of the three isolates had drug-resistant variants.

**Table 1:** *Mycobacterium tuberculosis* lineages, sub-lineages, and drug resistance type of the two elephant and one human isolates collected and sequenced in this study and identified using TB-Profiler v4.1.0.

Sample	Host	Lineage	Sub-lineage	Drug-resistance type
52G111	Human	Lineage 4	Lineage 4.1.2.1	Sensitive
PK	Elephant	Lineage 2	Lineage 2.2	Sensitive
DG	Elephant	Lineage 1	Lineage 1.2.2.2	Sensitive

#### 3.2.1. Whole genome and phylogenomic analysis

The mean coverage of the three *M. tuberculosis* genomes was 99.6%, while the mean depth was 55.67*x*, with the isolate DG having the lowest mean depth of 39*x* (Table 2). The PhaME pipeline generated a core genome alignment of ~200 kbp and a non-synonymous SNP alignment of 2,596 bp size, which included the elephant and human isolates and the genomes downloaded from the NCBI Sequence Read Archive database. The SNP alignment was used for phylogenomic analysis (Table 2).

**Table 2:** Size, coverage, and mean depth of the analysed *M. tuberculosis* genomes after merging of the Illumina short reads and Nanopore long reads.

Sample	Size (bp)	Genome coverage (%)	Mean depth (*x*)
52G111	4,411,532	99.59	60.13
DG	4,411,532	99.69	39.29
PK	4,411,532	99.51	67.58

**Figure 2:**
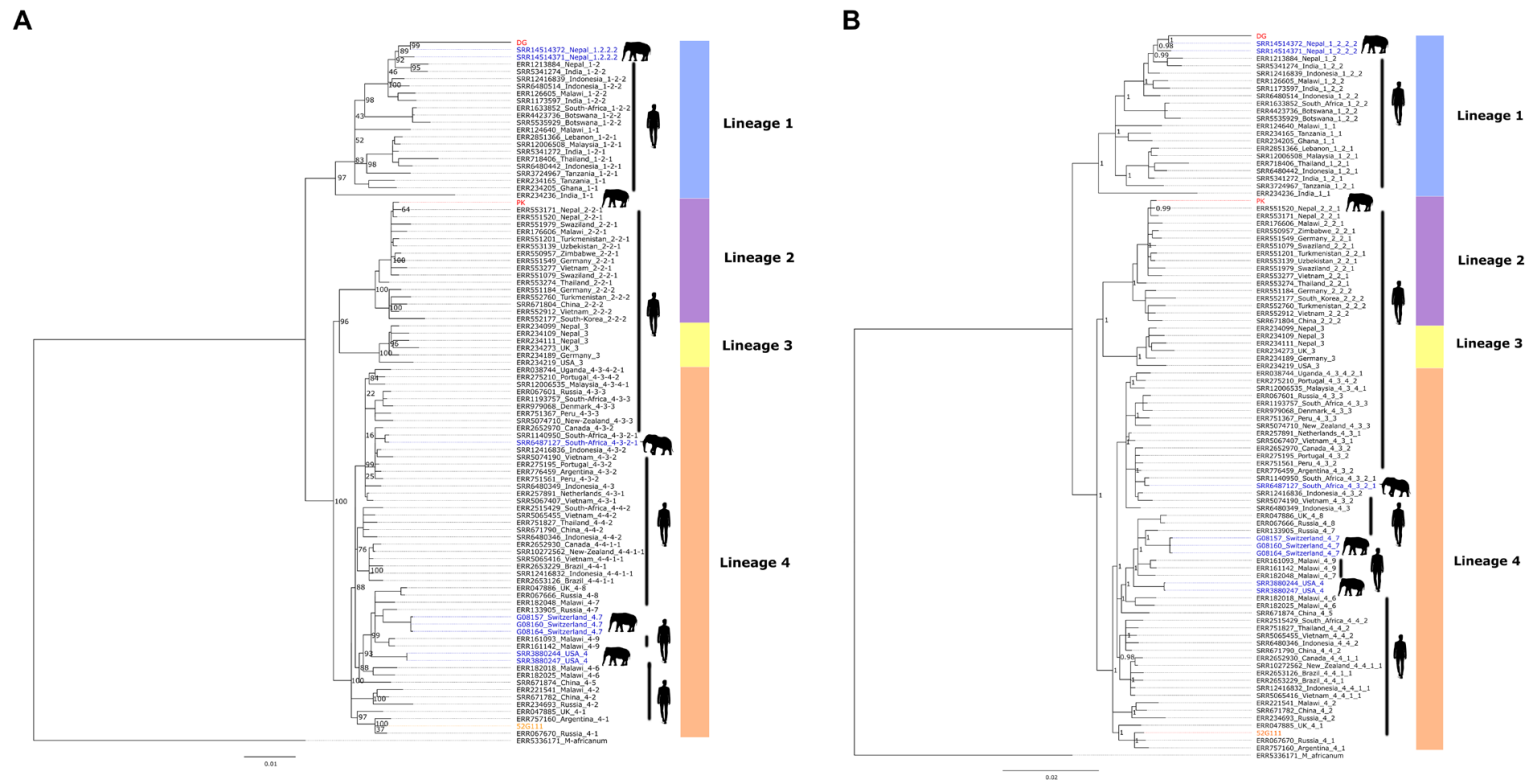
Phylogenomic trees constructed using the 2,596 bp non-synonymous SNP alignment comprising the three new *M. tuberculosis* genomes described in this study and the 94 genome sequences retrieved from GenBank: **(A)** IQTREE maximum likelihood tree, the values in the nodes are 500 bootstrap scores; **(B)** MrBayes Bayesian inference tree, the values in the nodes are posterior probability scores. The elephant (PK, DG) and human (52G111) samples generated in this study are shown in red and orange font, respectively, while the published elephant and human derived samples are shown in blue and black font, respectively.

The topology of both maximum-likelihood and Bayesian inference trees were similar, as shown in Figure 2. The phylogenomic analysis of *M. tuberculosis* sequences revealed that the two elephant-derived isolates, DG and PK, clustered into clades of two different lineages, lineage 1 and lineage 2, respectively. Similarly, the human-derived isolate, 52G111, clustered within into lineage 4 clade. This result is consistent with the lineages/sub-lineages defined by TB-Profiler. Sample DG is evolutionary closely related to two other published genomes of elephant-derived isolates sampled in Nepal (SRR14514371 and SRR14514372). These three elephant-derived sequences clustered together, in the same sub-clade, with published human-derived isolates sampled in Nepal (ERR1213884) and India (SRR5341274). Similarly, sample PK clustered closely in the same clade with published human-derived isolates sampled in Nepal (ERR553171 and ERR551520). The human sample 52G111 clustered in a clade together with other published human-derived samples from Argentina (ERR757160), Russia (ERR067670) and the United Kingdom (ERR047885). The topology of the tree also showed that previously published *M. tuberculosis* isolates derived from captive Asian elephants in the USA and Switzerland belonged to lineage 4 and clustered in a clade with human-derived isolates from nearby geographical regions. Similarly, an isolate originating from wild African elephants (SRR6487127) clustered very closely with a human-derived isolate (SRR1140950), both sampled in South Africa.

## 4. Discussion

In this study, we characterised elephant-derived *M. tuberculosis* isolates from two diseased animals (PK and DG), and a human-derived *M. tuberculosis* isolate (52G111) using a whole-genome sequencing approach combining Illumina short reads and Nanopore long reads, resulting in an average genome coverage of 99.6% with an average depth of 55.67*x*. Phylogenomic analyses were used to explore possible spillover of *M. tuberculosis* between humans and elephants. We also used an online-based TB profiling tool (TB-Profiler) to further identify lineages/sublineages of the sequenced isolates and to assess drug-resistant variants.

The two elephant-derived *M. tuberculosis* isolates appear to be of human origin based on both the TB-Profiler result and the phylogenomic trees. These strains are evolutionarily closely related to previous human isolates sampled in Nepal (Figure 1), suggesting a bidirectional transmission from humans to elephants. This may be due to the high prevalence of TB in humans and the continuous exposure of elephants to their handlers, the mahouts, rather than to the sporadic contact with tourists (48). Despite living in the same locality, the two elephant-derived *M. tuberculosis* isolates clustered in different clades of TB lineages, suggesting a complex and diverse *M. tuberculosis* transmission dynamics in the region. There must be multiple sources of infection and cross-species (human-mediated) transmission other than direct elephant-to-elephant transmission. This conclusion could be drawn from the fact that lineage 2 is the second most prevalent lineage in Nepal according to human samples from 2009 to 2010, while lineage 1 is the least prevalent according to a study conducted in 2018 (20). The possibility of bidirectional interspecies transmission of *M. tuberculosis* between humans and elephants has also been supported by previous reports (49–51). Our results reinforce previous evidence suggesting a close association between *M. tuberculosis* isolates from elephants and humans in Nepal using *M. tuberculosis* spoligotyping (15, 24), large sequence polymorphism (24) or whole genome data (25).

Tuberculosis is known as a re-emerging disease, but recently it has been considered a zooanthroponosis (52). The observed cases of zooanthroponosis have implications for global and national elephant conservation. Human tuberculosis already contributes to a huge annual burden of morbidity and mortality, and cases are concentrated in countries such as Nepal where poverty and high population density overlap. Multi-drug resistant *M. tuberculosis* is an additional concern, and the global burden of zoonotic TB is increasing due to the uncontrolled use of antibiotics in animals (53). Zoonotic TB is severely under-reported due to diagnostic challenges and inadequate public health surveillance (54). However, this study attempts to fill this gap in Nepal. Furthermore, the risk factors prevalent in South Asian countries, including Nepal, such as high human-animal density, close and frequent contact with infected animals, inadequate disease control measures, consumption of unpasteurised milk and milk products (55), and use of elephants in tourism (56), explain the possibility of zoonotic TB cases in humans as well as zooanthroponosis in animals. Nevertheless, a serious risk factor analysis should be undertaken to better understand the complex and dynamic transmission of TB in Nepal.

In conclusion, the prospect of an elephant potentially acting as a carrier/reservoir for the transmission of drug-resistant *M. tuberculosis* as a result of human-animal interactions is a serious concern that may pose future risks not only to humans but also to TB control in livestock and wildlife (57). A One Health approach is fundamental to understanding the spillover or transmission dynamics of infectious diseases such as TB. Therefore, regular screening and detection of these infectious diseases in elephants, other wild and domestic animals, human populations and the environment is essential. The Food and Agriculture Organization of the United Nations (FAO) has already identified in its ‘Roadmap for Zoonotic Tuberculosis’ (58) a wide range of actions involving a wide range of stakeholders to reduce the risk of transmission, thereby bringing economic benefits and improvements in animal welfare. The One Health Strategic Framework 2019 has already been approved in Nepal (59). In Nepal, implementation of the One Health framework appears to be possible only with a focus on zoonoses and zooanthroponosis. Therefore, it should be monitored through surveillance systems and better screening and diagnostic strategies using information on circulating *M. tuberculosis* genotypes as identified in this study. However, to limit transmission of the pathogen, oral BCG administration provides significant protection against human (60) and animal (61) TB in addition to surveillance prevention strategies. In the context of Nepal, regular *M. tuberculosis* screening of all captive elephants and people in close contact with them, especially mahouts, should be promoted to control and prevent the spread of TB. This will also help prevent the spread of TB to other endangered species in the forest (62). In addition, good practices and guidelines for tourism-related activities involving direct human-animal interactions, such as mahout-elephant interaction, should be implemented as a preventive measure.

## Data availability statement

The datasets presented in this study can be found in online repositories. The raw FASTQ genome data is available in NCBI SRA database under BioProject ID: PRJNA884899.

## Ethics statement

The studies involving human participants were reviewed and approved by Ethical approval for the study was obtained from Nepal Ethical Review Board, Nepal Health Research Council (IRC number 312/2018). The patients/participants provided their written informed consent to participate in this study. Ethical review and approval was not required for the animal study because we took the samples from natural deceased animals.

## Author contributions

RMR and JQ prepared the overall concept and design. RN, AG, RR, and PM performed the bioinformatics analysis, lab processing, and analysis. AP and NS contributed to the literature review. AS and RMR were involved in sample collection. RMR, NS, CG, DK, PA, JF, and JQ prepared the main writing part of the manuscript and methodology design. All authors contributed to the article and approved the submitted version.

## Funding

This work was supported by National Funds through FCT-Fundação para a Ciência e a Tecnologia in the scope of the project UIDP/50027/2020.

## Conflict of interest

The authors declare that the research was conducted in the absence of any commercial or financial relationships that could be construed as a potential conflict of interest.

## Publisher’s note

All claims expressed in this article are solely those of the authors and do not necessarily represent those of their affiliated organizations, or those of the publisher, the editors and the reviewers. Any product that may be evaluated in this article, or claim that may be made by its manufacturer, is not guaranteed or endorsed by the publisher.
