# Supplementary material for: Phylogenomic analysis supports Mycobacterium tuberculosis transmission between humans and elephants
Source: Front Vet Sci. 2023 May 26;10:1133823. doi: 10.3389/fvets.2023.1133823 (PMC10250650; doi:10.3389/fvets.2023.1133823)
Supplement: Supplementary file 1 [file Table_1.DOCX]

**Supplementary information**

Table S1: Detailed information on the 94 whole genomes retrieved from GenBank and used in this study.

| S.N. | Accession | Country | Region | Year | Lineage | Sub-lineage | Reference | Host species | Sequence ID |
| --- | --- | --- | --- | --- | --- | --- | --- | --- | --- |
| 1 | ERR124640 | Malawi | Malawi | NA | 1 | 1.1 | Guerra-Assunção_eLife_2015 | Human | ERR124640_Malawi_1-1 |
| 2 | ERR234236 | India | Southern Asia | NA | 1 | 1.1 | Comas_NatGenet_2013 | Human | ERR234236_India_1-1 |
| 3 | ERR234165 | Tanzania | Eastern Africa | NA | 1 | 1.1 | Comas_NatGenet_2013 | Human | ERR234165_Tanzania_1-1 |
| 4 | ERR234205 | Ghana | Eastern Africa | NA | 1 | 1.1 | Comas_NatGenet_2013 | Human | ERR234205_Ghana_1-1 |
| 5 | ERR2851366 | Lebanon | Eurasia | 2016 | 1 | 1.2.1 | NA | Human | ERR2851366_Lebanon_1-2-1 |
| 6 | ERR718406 | Thailand | Southeast Asia | 2003 | 1 | 1.2.1 | NA | Human | ERR718406_Thailand_1-2-1 |
| 7 | SRR12006508 | Malaysia | Southeast Asia | 2016 | 1 | 1.2.1 | NA | Human | SRR12006508_Malaysia_1-2-1 |
| 8 | SRR3724967 | Tanzania | Africa | 2013 | 1 | 1.2.1 | NA | Human | SRR3724967_Tanzania_1-2-1 |
| 9 | SRR5341272 | India | South Asia | 2015 | 1 | 1.2.1 | NA | Human | SRR5341272_India_1-2-1 |
| 10 | SRR6480442 | Indonesia | Southeast Asia | 2014 | 1 | 1.2.1 | NA | Human | SRR6480442_Indonesia_1-2-1 |
| 11 | ERR126605 | Malawi | Africa | 2007 | 1 | 1.2.2 | NA | Human | ERR126605_Malawi_1-2-2 |
| 12 | ERR1633852 | South Africa | Africa | 2010 | 1 | 1.2.2 | NA | Human | ERR1633852_South Africa_1-2-2 |
| 13 | ERR4423736 | Botswana | Africa | 2013 | 1 | 1.2.2 | NA | Human | ERR4423736_Botswana_1-2-2 |
| 14 | SRR1173597 | India | South Asia | 2001 | 1 | 1.2.2 | NA | Human | SRR1173597_India_1-2-2 |
| 15 | SRR12416839 | Indonesia | Southeast Asia | 2017 | 1 | 1.2.2 | NA | Human | SRR12416839_Indonesia_1-2-2 |
| 16 | SRR5341274 | India | South Asia | 2015 | 1 | 1.2.2 | NA | Human | SRR5341274_India_1-2-2 |
| 17 | SRR5535929 | Botswana | Africa | 2013 | 1 | 1.2.2 | NA | Human | SRR5535929_Botswana_1-2-2 |
| 18 | SRR6480514 | Indonesia | Southeast Asia | 2015 | 1 | 1.2.2 | NA | Human | SRR6480514_Indonesia_1-2-2 |
| 19 | ERR176606 | Malawi | Malawi | NA | 2 | 2.2.1 | Guerra-Assunção_eLife_2015 | Human | ERR176606_Malawi_2-2-1 |
| 20 | ERR550957 | Zimbabwe | Eastern Africa | NA | 2 | 2.2.1 | Merker_NatGenet_2015 | Human | ERR550957_Zimbabwe_2-2-1 |
| 21 | ERR553277 | Vietnam | Vietnam | NA | 2 | 2.2.1 | Merker_NatGenet_2015 | Human | ERR553277_Vietnam_2-2-1 |
| 22 | ERR553139 | Uzbekistan | Central Asia | NA | 2 | 2.2.1 | Merker_NatGenet_2015 | Human | ERR553139_Uzbekistan_2-2-1 |
| 23 | ERR551201 | Turkmenistan | Central Asia | NA | 2 | 2.2.1 | Merker_NatGenet_2015 | Human | ERR551201_Turkmenistan_2-2-1 |
| 24 | ERR553274 | Thailand | South-Eastern Asia | NA | 2 | 2.2.1 | Merker_NatGenet_2015 | Human | ERR553274_Thailand_2-2-1 |
| 25 | ERR551079 | Swaziland | Southern Africa | NA | 2 | 2.2.1 | Merker_NatGenet_2015 | Human | ERR551079_Swaziland_2-2-1 |
| 26 | ERR551979 | Swaziland | Southern Africa | NA | 2 | 2.2.1 | Merker_NatGenet_2015 | Human | ERR551979_Swaziland_2-2-1 |
| 27 | ERR551549 | Germany | Northern Europe | NA | 2 | 2.2.1 | Merker_NatGenet_2015 | Human | ERR551549_Germany_2-2-1 |
| 28 | ERR552912 | Vietnam | Vietnam | NA | 2 | 2.2.2 | Merker_NatGenet_2015 | Human | ERR552912_Vietnam_2-2-2 |
| 29 | ERR552760 | Turkmenistan | Central Asia | NA | 2 | 2.2.2 | Merker_NatGenet_2015 | Human | ERR552760_Turkmenistan_2-2-2 |
| 30 | ERR552177 | South Korea | Eastern Asia | NA | 2 | 2.2.2 | Merker_NatGenet_2015 | Human | ERR552177_South Korea_2-2-2 |
| 31 | ERR551184 | Germany | Northern Europe | NA | 2 | 2.2.2 | Merker_NatGenet_2015 | Human | ERR551184_Germany_2-2-2 |
| 32 | SRR671804 | China | China | NA | 2 | 2.2.2 | Zhang_NatGenet_2013 | Human | SRR671804_China_2-2-2 |
| 33 | ERR234189 | Germany | Europe | NA | 3 | 3 | Comas et al. 2013 | Human | ERR234189_Germany_3 |
| 34 | ERR234219 | USA | NA | NA | 3 | 3 | Comas et al. 2013 | Human | ERR234219_USA_3 |
| 35 | ERR234273 | UK | Northern Europe | NA | 3 | 3 | Comas et al. 2013 | Human | ERR234273_UK_3 |
| 36 | ERR047885 | UK | Northern Europe | NA | 4 | 4.1 | Casali_NatGenet_2014 | Human | ERR047885_UK_4-1 |
| 37 | ERR067670 | Russia | Russia | NA | 4 | 4.1 | Casali_NatGenet_2014 | Human | ERR067670_Russia_4-1 |
| 38 | ERR757160 | Argentina | Argentina | NA | 4 | 4.1 | Eldholm_NatComms_2015 | Human | ERR757160_Argentina_4-1 |
| 39 | ERR234693 | Russia | Russia | NA | 4 | 4.2 | Casali_NatGenet_2014 | Human | ERR234693_Russia_4-2 |
| 40 | ERR221541 | Malawi | Malawi | NA | 4 | 4.2 | Guerra-Assunção_eLife_2015 | Human | ERR221541_Malawi_4-2 |
| 41 | SRR671782 | China | China | NA | 4 | 4.2 | Zhang_NatGenet_2013 | Human | SRR671782_China_4-2 |
| 42 | SRR6480349 | Indonesia | Southeast Asia | 2014 | 4 | 4.3 | NA | Human | SRR6480349_Indonesia_4-3 |
| 43 | SRR671874 | China | China | NA | 4 | 4.5 | Zhang_NatGenet_2013 | Human | SRR671874_China_4-5 |
| 44 | ERR182018 | Malawi | Malawi | NA | 4 | 4.6 | Guerra-Assunção_eLife_2015 | Human | ERR182018_Malawi_4-6 |
| 45 | ERR182025 | Malawi | Malawi | NA | 4 | 4.6 | Guerra-Assunção_eLife_2015 | Human | ERR182025_Malawi_4-6 |
| 46 | ERR133905 | Russia | Russia | NA | 4 | 4.7 | Casali_NatGenet_2014 | Human | ERR133905_Russia_4-7 |
| 47 | ERR182048 | Malawi | Malawi | NA | 4 | 4.7 | Guerra-Assunção_eLife_2015 | Human | ERR182048_Malawi_4-7 |
| 48 | ERR047886 | UK | Northern Europe | NA | 4 | 4.8 | Casali_NatGenet_2014 | Human | ERR047886_UK_4-8 |
| 49 | ERR067666 | Russia | Russia | NA | 4 | 4.8 | Casali_NatGenet_2014 | Human | ERR067666_Russia_4-8 |
| 50 | ERR161093 | Malawi | Malawi | NA | 4 | 4.9 | Guerra-Assunção_eLife_2015 | Human | ERR161093_Malawi_4-9 |
| 51 | ERR161142 | Malawi | Malawi | NA | 4 | 4.9 | Guerra-Assunção_eLife_2015 | Human | ERR161142_Malawi_4-9 |
| 52 | ERR257891 | Netherlands | Europe | 1994 | 4 | 4.3.1 | NA | Human | ERR257891_Netherlands_4-3-1 |
| 53 | SRR5067407 | Vietnam | Southeast Asia | 2010 | 4 | 4.3.1 | NA | Human | SRR5067407_Vietnam_4-3-1 |
| 54 | ERR2652970 | Canada | North America | 2000 | 4 | 4.3.2 | NA | Human | ERR2652970_Canada_4-3-2 |
| 55 | ERR275195 | Portugal | Europe | 2009 | 4 | 4.3.2 | NA | Human | ERR275195_Portugal_4-3-2 |
| 56 | ERR751561 | Peru | South America | 2011 | 4 | 4.3.2 | NA | Human | ERR751561_Peru_4-3-2 |
| 57 | ERR776459 | Argentina | South America | 2005 | 4 | 4.3.2 | NA | Human | ERR776459_Argentina_4-3-2 |
| 58 | SRR12416836 | Indonesia | Southeast Asia | 2017 | 4 | 4.3.2 | NA | Human | SRR12416836_Indonesia_4-3-2 |
| 59 | SRR5074190 | Vietnam | Southeast Asia | 2009 | 4 | 4.3.2 | NA | Human | SRR5074190_Vietnam_4-3-2 |
| 60 | SRR1140950 | South Africa | Africa | 2013 | 4 | 4.3.2.1 | NA | Human | SRR1140950_South Africa_4-3-2-1 |
| 61 | ERR067601 | Russia | Eurasia | 2008 | 4 | 4.3.3 | NA | Human | ERR067601_Russia_4-3-3 |
| 62 | ERR1193757 | South Africa | Africa | NA | 4 | 4.3.3 | NA | Human | ERR1193757_South Africa_4-3-3 |
| 63 | ERR751367 | Peru | South America | 2009 | 4 | 4.3.3 | NA | Human | ERR751367_Peru_4-3-3 |
| 64 | ERR979068 | Denmark | Europe | 1994 | 4 | 4.3.3 | NA | Human | ERR979068_Denmark_4-3-3 |
| 65 | SRR5074710 | New Zealand | Pacific | 2007 | 4 | 4.3.3 | NA | Human | SRR5074710_New Zealand_4-3-3 |
| 66 | SRR12006535 | Malaysia | Southeast Asia | 2013 | 4 | 4.3.4.1 | NA | Human | SRR12006535_Malaysia_4-3-4-1 |
| 67 | ERR275210 | Portugal | Europe | 2010 | 4 | 4.3.4.2 | NA | Human | ERR275210_Portugal_4-3-4-2 |
| 68 | ERR038744 | Uganda | Africa | 2004 | 4 | 4.3.4.2.1 | NA | Human | ERR038744_Uganda_4-3-4-2-1 |
| 69 | ERR2653229 | Brazil | South America | 2010 | 4 | 4.4.1 | NA | Human | ERR2653229_Brazil_4-4-1 |
| 70 | ERR2652930 | Canada | North America | 1997 | 4 | 4.4.1.1 | NA | Human | ERR2652930_Canada_4-4-1-1 |
| 71 | ERR2653126 | Brazil | South America | 2009 | 4 | 4.4.1.1 | NA | Human | ERR2653126_Brazil_4-4-1-1 |
| 72 | SRR10272562 | New Zealand | Pacific | 2001 | 4 | 4.4.1.1 | NA | Human | SRR10272562_New Zealand_4-4-1-1 |
| 73 | SRR12416832 | Indonesia | Southeast Asia | 2017 | 4 | 4.4.1.1 | NA | Human | SRR12416832_Indonesia_4-4-1-1 |
| 74 | SRR5065416 | Vietnam | Southeast Asia | 2011 | 4 | 4.4.1.1 | NA | Human | SRR5065416_Vietnam_4-4-1-1 |
| 75 | ERR2515429 | South Africa | Africa | NA | 4 | 4.4.2 | NA | Human | ERR2515429_South Africa_4-4-2 |
| 76 | ERR751827 | Thailand | Southeast Asia | NA | 4 | 4.4.2 | NA | Human | ERR751827_Thailand_4-4-2 |
| 77 | SRR5065455 | Vietnam | Southeast Asia | 2010 | 4 | 4.4.2 | NA | Human | SRR5065455_Vietnam_4-4-2 |
| 78 | SRR6480346 | Indonesia | Southeast Asia | 2014 | 4 | 4.4.2 | NA | Human | SRR6480346_Indonesia_4-4-2 |
| 79 | SRR671790 | China | East Asia | NA | 4 | 4.4.2 | NA | Human | SRR671790_China_4-4-2 |
| 80 | ERR234099 | Nepal | NA | NA | 3 | 3 | Comas et al. 2013 | Human | ERR234099_Nepal_3 |
| 81 | ERR234109 | Nepal | NA | NA | 3 | 3 | Comas et al. 2013 | Human | ERR234109_Nepal_3 |
| 82 | ERR234111 | Nepal | NA | NA | 3 | 3 | Comas et al. 2013 | Human | ERR234111_Nepal_3 |
| 83 | ERR1213884 | Nepal | Southern Asia | NA | 1 | 1.2 | Phelan_BMCMedicine_2016 | Human | ERR1213884_Nepal_1-2 |
| 84 | ERR551520 | Nepal | Southern Asia | NA | 2 | 2.2.1 | Merker_NatGenet_2015 | Human | ERR551520_Nepal_2-2-1 |
| 85 | ERR553171 | Nepal | Southern Asia | NA | 2 | 2.2.1 | Merker_NatGenet_2015 | Human | ERR553171_Nepal_2-2-1 |
| 86 | SRR14514372 | Nepal | NA | NA | 1 | 1.2.2.2 | https://journals.asm.org/doi/10.1128/MRA.00614-21 | Asian elephant  (*Elephas maximus*) | SRR14514372_Nepal_1-2-2-2 |
| 87 | SRR14514371 | Nepal | NA | NA | 1 | 1.2.2.2 | https://journals.asm.org/doi/10.1128/MRA.00614-21 | Asian elephant  (*Elephas maximus*) | SRR14514371_Nepal_1-2-2-2 |
| 88 | SRR6487127 | South Africa | NA | NA | 4 | 4.3.2.1 | https://www.frontiersin.org/articles/10.3389/fvets.2019.00018/full | African savanna elephant (*Loxodonta Africana*) | SRR6487127_South Africa_4-3-2-1 |
| 89 | G08157 | Switzerland | NA | NA | 4 | 4.7 | DOI:10.1038/s41598-017-15278-9 | Asian elephant  (*Elephas maximus*) | G08157_Switzerland_4-7 |
| 90 | G08164 | Switzerland | NA | NA | 4 | 4.7 | DOI:10.1038/s41598-017-15278-9 | Asian elephant  (*Elephas maximus*) | G08164_Switzerland_4-7 |
| 91 | G08160 | Switzerland | NA | NA | 4 | 4.7 | DOI:10.1038/s41598-017-15278-9 | Asian elephant  (*Elephas maximus*) | G08160_Switzerland_4-7 |
| 92 | SRR3880244 | USA | NA | NA | 4 | 4 | http://dx.doi.org/10.3201/eid2303.160726 | Asian elephant  (*Elephas maximus*) | SRR3880244_USA_4 |
| 93 | SRR3880247 | USA | NA | NA | 4 | 4 | http://dx.doi.org/10.3201/eid2303.160726 | Asian elephant  (*Elephas maximus*) | SRR3880247_USA_4 |
| 94 | ERR5336171 | Outgroup | NA | NA | *M. africanum* | NA | NA | NA | NA |
